# Simulating the biochemical environment for infant intestinal digestion: key challenges and recommendations

**DOI:** 10.3389/fnut.2026.1826318

**Published:** 2026-06-24

**Authors:** Linda Nezbedova, Aiqian Ye, Alejandra Acevedo-Fani, Louise Brough, Harjinder Singh, Debashree Roy

**Affiliations:** 1 Riddet Institute, Massey University, Palmerston North, New Zealand; 2 School of Food Technology and Natural Sciences, Massey University, Palmerston North, New Zealand

**Keywords:** breast milk, digestion, *in vitro*, infant, small intestine

## Abstract

Digestion in the small intestine is a crucial step in the breakdown and absorption of nutrients from human milk. *In vitro* models of intestinal digestion are commonly employed to investigate the digestion behaviour of breast milk nutrients. Accurate replication of the infant small intestine requires a detailed understanding of its biochemical environment. This review summarises current knowledge on small intestinal digestion in infants, integrating evidence from both human studies and *in vitro* intestinal digestion models. Key challenges in simulating the infant small intestinal conditions, including motility, intestinal fluid dynamics and composition, enzymatic activity, and absorption are discussed. This literature review also provides recommendations for enhancing the physiological relevance of *in vitro* small intestinal digestion systems for studying intestinal digestion in early infancy.

## 1 Introduction

Early infancy (up to six months) is a critical period for growth and development, yet the processes by which nutrients from breast milk are digested and absorbed are not fully understood. Conducting studies in infants or animal models is challenging due to ethical and technical constraints, high costs, and interindividual variability. Thus, *in vitro* digestion studies have become a valuable tool for investigating infant digestion (1). Designing these studies requires careful selection of parameters that accurately reflect the infant gastrointestinal tract physiology. However, many existing physiological data are outdated and often derived from preterm infants, providing an incomplete foundation for defining appropriate *in vitro* digestion conditions.

The main source of nutrition for infants up to six months is breast milk, with infant formula as an alternative when breastfeeding is not possible or chosen (2). Digestion begins in the oral cavity, but swallowing predominates over mastication in infants when consuming liquids (3). Therefore, the oral phase is often omitted in *in vitro* models of breast milk digestion. Simulating infant gastric digestion presents its own challenges, which has been recently reviewed by Wang et al. (4). The intestinal phase, however, remains the least defined. In the small intestine the majority of macronutrient breakdown and nutrient absorption occurs (5). The infant intestine undergoes rapid postnatal development, with structure and function still maturing (6). It has been reported that early feeding promotes epithelial growth in infants (7, 8). For example, studies in piglets (animal model for infant) show colostrum (first breast milk) intake increased intestinal size and cellular content within 24 h (9), and the small intestine continued to elongate in the first months of life (6, 10). Additionally, there are interindividual developmental variations among infants. These dynamic changes, combined with limited data on key parameters such as intestinal motility, pH, secretions, and nutrient absorption make it challenging to design physiologically relevant *in vitro* models for infant intestinal digestion.

This mini review examines and synthesises current knowledge on the biochemical environment of infant small intestinal digestion. It integrates findings from studies in human infants and their translation into *in vitro* models, with a focus on breast milk digestion in healthy term infants up to six months of age. By highlighting the main challenges and providing recommendations, we aim to advance the design of physiologically relevant *in vitro* small intestinal protocols for studying breast milk digestion in this population.

## 2 Challenges and recommendations in simulating infant small intestinal digestion with an *in vitro* model

*In vitro* intestinal digestion models are generally categorised as static or dynamic depending on how physiological conditions are represented during digestion. Static models apply fixed experimental parameters and provide simplified and reproducible models for assessing nutrient breakdown under standardised conditions. Dynamic models aim to better mimic the nature of gastrointestinal digestion by incorporating time-dependent changes such as gradual secretion of digestive fluid and controlled transit between compartments, as implemented in infant adapted systems such as DIDGI (11, 12).

**Figure 1:**
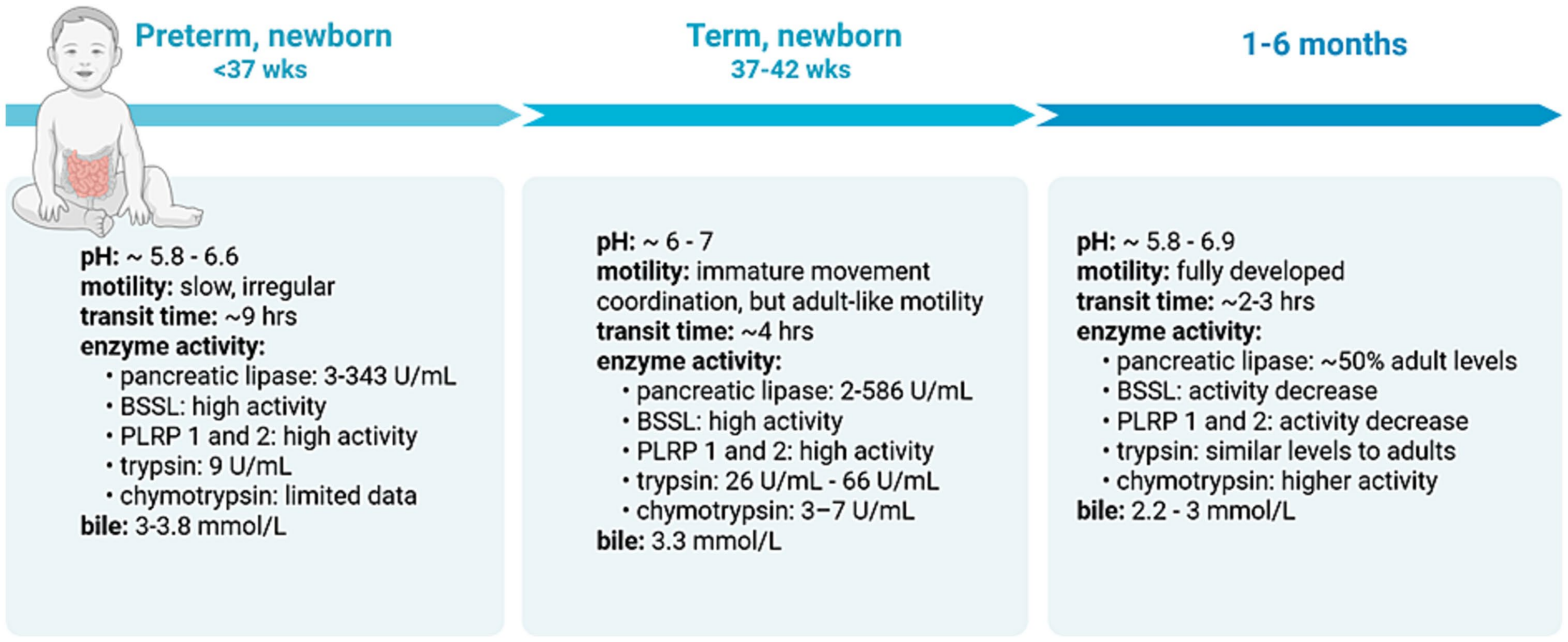
Developmental changes in small intestinal digestion conditions in preterm infants, term infants, and infants up to six months of age. Data are based on previous *in vivo* studies described in this review. Created with BioRender.com.

Current *in vitro* studies use mainly static models to simulate infant intestinal digestion (13–19), with few dynamic models employed (11, 12, 20, 21). Although the infant small intestine at 12 weeks of gestation (5, 22) comprises three anatomical parts; duodenum, jejunum, and ileum, static *in vitro* models are usually limited to a single segment. These models typically simulate the duodenum, as it is the main site of pancreatic juice secretion and as a first place of nutrient absorption. Applying physiologically relevant *in vitro* digestion models requires accounting for multiple factors, such as intestinal motility and transit time, intestinal fluid dynamics, intestinal secretions, enzyme activity, bile concentrations, intestinal acidity and absorption. These factors vary with gestational maturity and infant’s age and develop over time to meet the nutritional needs of the growing infant as depicted in Figure 1. Such developmental differences affect nutrient digestion and absorption and emphasise the need for *in vitro* models that accurately reflect infant physiology to provide meaningful insights into digestion. Table 1 summarises physiological data on key small intestinal digestion conditions derived from human infants and parameters used in current dynamic and static *in vitro* intestinal digestion models focusing on infants up to six months of age. Data for older infants are included for certain parameters, where data for infants below six months are limited. These parameters with respect to key challenges and recommendations are further discussed in the following sections.

**Table 1:** Comparative overview of the main small intestinal digestion conditions reported in published studies in human infants and digestion parameters used in current *in vitro* small intestinal models.

	Clinical studies			*In vitro* digestion studies		
Parameter	Value	Age*	Ref	Value	Model	Ref
Transit time	3 h	GA 26–33 wks	(31)	1 h	Static	(13, 16, 18)
	9 h	GA 32 wks	(30)	2 h	Static Dynamic	(15, 17, 19, 54) (20)
	4 h	40 wks	(30)	3 h	Static Dynamic	(12) (21)
Intestinal pH	5.4–8.4 (duodenum)	GA 27–41 wks	(96)	6.2	Static Dynamic	(12) (21)
	6.6 (duodenum)	34 wks, 8 days −19 yrs., up to 4 yrs	(99–101)	6.5	Static Dynamic	(14, 54) (11)
	5.8–6.9	1–3 mos	(102)	6.6	Static	(13)
	6–7 (duodenum)	1 mo–12 yrs	(122)	7.5	Static	(17)
				8	Static	(15)
Intestinal fluid flow	8.3 μL/min/kg	1 wk	(32)	0.75 mL/min	Dynamic	(12, 21)
				0.50 mL/min	Dynamic	(11)
Intestinal fluid electrolyte composition	Na^+^ 164 mM, K^+^ 10 mM, HCO_3_^−^ 85 mM, Ca^++^ 3 mM	1 wk	(32)	NaCl 164 mM, KCl 10 mM, NaHCO_3_ 85 mM, CaCl_2_ 3 mM	Static Dynamic	(13, 14) (11)
	Na^+^ 82–115 mM, K^+^ 7–17 mM, Cl^−^ 114-134 mM	Newborn	(41)			
Pancreatic lipase activity	15.9 U/L (fasted)	GA 33, 36 wks	(46)	90 U/mL SIF	Static	(13, 19)
	20–233 U/mL (fasted)	3–15 days	(48)	200 U/mL SIF	Static	(15, 17)
	77.4 U/kg	GA 32–34 wks	(47)			
	328.8 U/kg	GA 32–34 wks, 1 wk	(47)			
	39.9 U/kg	1 wk	(47)			
	149–586 U/mL (fasted)	1–25 days	(123)			
Colipase activity	5.1 U/kg/min	6 mos	(52)	2:1 (V/V) to pancreatic lipase	Static	(54)
BSSL	Active (values not specified)	12–21 wks	(63)	Not used		
PLRP 1,2	100% activity	42 wks	(57)	Not used		
Phospholipase A2	No Activity	up to 6 mos	(65)	Not used		
Trypsin activity	8.5 U/mL (fasted)	GA 33, 36 wks	(46)	3.45 U/mg protein	Static	(15, 16, 74)
	96.6 μg/mL (postprandial)	1 wk	(47)	10 times lower than adults	Static	(14)
	119.5 μg/mL (fasted)	3–15 days	(48)			
	93.8 μg/mL (postprandial)	3–15 days	(48)			
	90–92 μg/mL	3 and 5 mos	(71)			
α-chymotrypsin activity	6.6 nmol/min/mL (fasted)	1 mo	(69)	0.04 U/mg protein	Static	(15, 16, 74)
	5.3 nmol/min/mL (fasted)	Newborn	(69)	10 times lower than adults	Static	(14)
Elastase	0.6 μg/mL	GA 23–30 wks	(78)	not used		
Carboxypeptidase	2.9 nmol/min/mL	1 mo	(69)	not used		
Amylase	No activity	Term, newborn	(69)	75 U/mL	Dynamic	(20)
	No activity	Preterm, newborn	(72)			
	1.2–5.99 U/mL	Newborn	(47)			
Lactase	25 U/g protein	2–5 mos	(87)	Not used		
	7.9–19 μmol/g	Newborn	(86)			
Sphingomyelinase	13 nmol/h/mg protein	Newborn	(91)	Not used		

* Where gestational age (GA) is specified, the data refers to preterm infants, otherwise, the data represents term infants Preterm infants are born before 37 weeks of gestation, term infants are born at/after 37 weeks of gestation. Data for infants up to six months is primarily used, but studies reporting age range from infants to young children were used when age specific data could not be separated.

### 2.1 Intestinal motility and transit time

Intestinal motility refers to coordinated movements of the intestine that propel digesta along the gastrointestinal tract (23). The first intestinal motion occurs within the 48 h after birth (24), although evidence is limited. By 40 weeks gestational age (GA), duodenal motility reaches a level comparable to adults (25). Preterm infants display immature motility patterns that gradually mature with GA, as reported by Berseth (26) and Amarnath et al. (27). This immaturity is coupled with the rapid anatomical development of the small intestine. Intestinal length increases substantially during gestation from 5 to 40 weeks GA (28). A study based on autopsy data reported intestinal lengths of approximately 125 cm at 20 weeks GA, 200 cm at 30 weeks GA, and 275 cm at term (29), which relative to body size, is a markedly longer small intestine compared to adults. The long length of the small intestine combined with immature motility in preterm infants contributes to slower intestinal transit, which decreases from approximately nine hours at 32 weeks GA to around four hours at 40 weeks GA (30). Bodé et al. (31) reported transit time in preterm infants (28.9 weeks GA) to be three hours. Different intestinal digestion duration was used in *in vitro* digestion studies starting from one (13, 16, 18), two and three (12, 21) hours. Given full motility development by 40 weeks GA, a two-hour intestinal phase is a reasonable approximation for infant *in vitro* intestinal models.

### 2.2 Intestinal fluid flow and intestinal content

Upon entry into the small intestine, gastric digesta is further diluted by intestinal secretions. One study reported intestinal fluid flow of 8.3 μL/min/kg in one week old term infant (32). Dynamic *in vitro* digestion models used different intestinal fluid flow rates, including 0.75 mL/min (11, 21) and 0.85 mL/min (13), based on previous *in vitro* dynamic digestion model (33) and study in piglets (34). Physiological data on dilution of meal with gastric chyme in the small intestine are limited in infants. *In vitro* digestion studies commonly mimic physiological dilution by using a 39:61 ratio of meal to simulated fluid (13, 35), based on values measured at 78 min of digestion in the DIDGI dynamic intestinal model and supported by piglet data (11). This corresponds to a dilution factor of approximately 1.6. Considering the limited availability of data, a dilution factor of about 1.6 is a reasonable approximate. Interestingly, this ratio aligns with a gastric chyme to intestinal secretion ratio of 1.6 reported at half gastric emptying time in healthy adults (36).

### 2.3 Infant intestinal secretions

The intestinal fluid is excreted in the duodenum via pancreas, gallbladder and different intestinal cells, such as, e.g., Goblet cells (37). The intestinal fluid is composed of electrolytes, enzymes, bile and water and create suitable environment for the digestion of the food, but also food transport and absorption (38). Developmental changes in the enzyme activities and bile concentration are described in Figure 1. It is important to note that a limitation in reviewing enzyme activities is the lack of standardisation in measuring and reporting units across previously reported studies, which complicates direct comparisons and highlights the need for harmonised protocols as suggested by INFOGEST (39). The electrolyte composition and intestinal enzyme activities are further discussed in the following sections.

#### 2.3.1 Electrolyte composition

Electrolytes in the intestinal fluid maintain osmotic balance and facilitate nutrient transport and absorption (40). In newborns with intestinal obstruction, gastrointestinal fluid contained 82–115 mM sodium (Na^+^), 7–17 mM potassium (K^+^), and 114–134 mM chloride (Cl^−^) (41). In one week old term infants, intestinal fluid contained 164 mM sodium (Na^+^), 10 mM potassium (K^+^), 3 mM calcium (Ca^++^), and 85 mM bicarbonate (HCO_3_^−^) (32). These concentrations are commonly used to prepare simulated intestinal fluid (SIF) in infant *in vitro* digestion studies (13, 14), with electrolytes added as chloride salts except bicarbonate, which is added as the sodium salt. However, a key limitation is the lack of data beyond the newborn stage, making it challenging to represent intestinal conditions across infancy.

#### 2.3.2 Intestinal lipolytic enzymes

Lipids from breast milk provide over half of infant’s energy intake (42). In early infancy, lipid digestion in the small intestine depends on enzymes distinct from those in adults, primarily pancreatic lipase, pancreatic lipase-related proteins 1 and 2 (PLRP1 and PLRP2), bile salt-stimulated lipase (BSSL).

Pancreatic lipase catalyses the hydrolysis of dietary triglycerides into free fatty acids and monoglycerides, facilitating lipid digestion (43). However, activity of pancreatic lipase in infants is markedly lower than in adults and varies with GA (44). Pancreatic lipase reaches approximately 50% of adult activity by twelve months of age, while its activity remains relatively low during the first six months of life (44). In term newborns, fasted pancreatic lipase activity ranges from 2.2–586 U/mL, compared with 2.9–343 U/mL in preterm infants (24–34 weeks GA) (45, 46). After one week of feeding, preterm infants show higher activity than term infants (328.8 vs. 39.9 U/mL, fasted or postprandial state not specified) (47). Another study reported that pancreatic lipase activity in term infants decreases to 27–105 U/mL postprandially (48).

Most *in vitro* intestinal models use pancreatin as a source of pancreatic lipase (11–13, 19, 21), typically matching 90 U/mL SIF based on data in newborn infants (48). *In vitro* studies employing pancreatic lipase reported use of higher activity, 171 U/mL (20) and 200 U/mL (17) SIF. Since pancreatic lipase activity was reported to decrease postprandially to below approximately 100 U/mL (as mentioned above), an activity of 90 U/mL SIF, as reported in infants and used in some *in vitro* studies, appears to fall within a more physiologically relevant activity range for infant *in vitro* digestion studies.

Colipase is an essential cofactor for pancreatic lipase activity, enabling lipase adsorption to the lipid water interface (49). One study reported that pancreatic lipase was inactive without colipase (50), another study demonstrated that adding exogenous colipase to duodenal secretions from patients with pancreatic disease restored lipase activity, confirming its synergistic role (51). Pancreatic lipase and colipase have been shown to be present at approximately same activity levels in children from 0.5 to 16 years of age (52) and healthy adults (51, 53). Despite its importance in lipid digestion, infant *in vitro* models of small intestine typically use pancreatin, which contains colipase (but its activity is poorly defined), potentially underestimating pancreatic lipase activity. In *in vitro* adult intestinal models, which use individual pancreatic lipase, colipase is added in molar excess to lipase (1, 2, 54), which corresponds to a mass ratio of approximately 1:2 colipase to lipase (55). Given that pancreatic lipase requires colipase for its activity, similar ratio could be maintained in infant *in vitro* intestinal models.

While pancreatic lipase is the predominant lipolytic enzyme in adults, infant fat digestion depends more heavily on PLRP1 and 2 and BSSL (44, 56), which are active immediately after birth as demonstrated in human foetuses (57) and newborn rats (58). Suckling mice deficient in the PLRP2 suffered from fat malabsorption (59). PLRP2 was also identified to act together with pancreatic lipase to break down the breast milk fat (60), suggesting the role of the PLRP2 in fat metabolism. PLRP1 and 2 are expressed 16 weeks of GA and reach their peak activity around 42 weeks of GA as identified in the human foetus’s pancreatic tissue (57).

BSSL lipolytic activity is known to be dependent on the presence of bile salts (61). BSSL hydrolyses monoglycerides produced by other lipases (61). In an infant *in vitro* breast milk digestion study, gastric and pancreatic lipases hydrolysed about two-thirds of ester bonds, mainly forming monoglycerides and fatty acids, while BSSL further aided digestion of formed monoglycerides and the remaining tri- and diacylglycerols, achieving over 90% total hydrolysis (62). One study reported that BSSL was detectable in foetal pancreatic tissue at six weeks of GA, with higher levels observed in pancreatic juice from 12–21 weeks old foetuses compared to adults (63). However, it is known, that the majority of BSSL for infants is derived from breast milk (64), with only a small contribution from the infant’s exocrine system (61).

BSSL and PLRP1 and 2 appear to play a significant role in intestinal lipid digestion in newborns and early infancy. However, data on their specific activity remain limited and these enzymes are not commercially available, posing challenge for their inclusion in infant *in vitro* digestion models. Consequently, most *in vitro* digestion studies rely on pancreatic lipase as the primary lipolytic enzyme. To accurately mimic infant intestinal digestion in early infancy, there is a clear need to produce these enzymes for use in infant *in vitro* intestinal models evaluating digestion of breast milk lipids.

Another lipolytic enzyme, pancreatic phospholipase A2 (PLA2) appears to have low activity up to six months of age and its activity increase in the weaning stage (65) based on study in rats. Due to PLA2 low activity in early infancy and low commercial availability, this enzyme is usually omitted in *in vitro* digestion studies.

#### 2.3.3 Intestinal proteolytic enzymes

Proteins are another important macronutrient in human breast milk (66). The proteolysis of proteins from ingested food is initiated in the stomach, however, the major digestion occurs in the small intestine (1). The main proteolytic enzymes in the infant small intestine are trypsin and alpha-chymotrypsin (*α*-chymotrypsin).

Trypsin is secreted by the pancreas into duodenum and activated from its inactive precursor, trypsinogen (67). In term infants, trypsin activity is already detectable after birth (68), and increases during the first month of life (69) reaching levels comparable to those of adults by approximately six months of age (44, 70). In term infants, trypsin activity prior to the first feed has been reported at 66.1 μg/mL, decreasing to 26.3 μg/mL within 24 h after the first feed, and rising to 96.6 μg/mL at one week of age (47). Similarly, 3–15 days old term infants showed fasting activity of 119.5 μg/mL, declining to 93.8 μg/mL postprandially (48). Older infants had trypsin activity 90–92 μg/mL at three and five months (71). Trypsin activity of 8.5 U/mL was reported in preterm infants born at 33–36 weeks gestation (46). In general, preterm infants were showed to exhibit lower trypsin activity than term infants at birth (47), with reported postprandial values below 40 μg/mL, compared to above 40 μg/mL in term infants (72). However, in another study, the trypsin activity 24 h after the first feed in preterm infants was reported to be greater (43.1 μg/mL) than term infants (26.3 μg/mL) (47).

Most *in vitro* digestion studies use pancreatin as a source of trypsin, with trypsin activity commonly adjusted to match 16 U/mL in SIF based on Norman et al. (48). It is important to note that pancreatin does not fully dissolve in SIF and instead forms a suspension containing undissolved particles. Therefore, pancreatin should be fully dissolved prior to the experiment to reduce variations, as previously reported (73).

*In vitro* infant digestion studies using isolated trypsin report activities approximately ten times lower than the adult trypsin activity recommended by INFOGEST (200 U/mL SIF) (14), corresponding to around 20 U/mL in SIF for infant digestion (55). Other *in vitro* digestion studies report using 3.45 U/mg of protein (15, 16, 74), but it is unclear if this was based on protein content of meal or protein content of the gastric chyme subjected to intestinal digestion. Considering that human breast milk contains approximately 1 g protein/100 mL milk (75), trypsin activity matched to the meal protein content would be approximately 35 U/mL. Since trypsin activity is reported to decrease postprandially, aligning it with meal protein content is recommended. However, the most accurate approach will be to base the activity on the protein content of the emptied gastric chyme entering the small intestine. In cases where *in vitro* intestinal digestion experiments must be conducted immediately after gastric digestion and protein content measurement of the gastric chyme is not feasible, using an activity of 20 U/mL SIF is recommended, as it falls within the physiological values previously reported (45, 48).

Another proteolytic enzyme, *α*-chymotrypsin is activated from its inactive precursor by trypsin (76). There is limited data on α-chymotrypsin activity in infants. Reported concentrations include 86.5 μg/mL (postprandial) in infants aged 3–15 days (48) and 6.6 nmol/min/mL in a one-month-old infant in fasted state (69). A literature review reported the *α*-chymotrypsin activity in term infants to be 2.6–6.7 U/mL (45). *In vitro* studies based on Norman et al. (48) used *α*-chymotrypsin at ten-fold lower activity than adult levels (14), therefore about 5 U/mL (55), consistent with physiological activity reported in infants. Other *in vitro* intestinal models have applied *α*-chymotrypsin at 0.04 U/mg of protein (15, 16, 74). When pancreatin is used, α-chymotrypsin activity is typically not reported, as pancreatin is standardised to trypsin activity. Despite limited data on α-chymotrypsin activity in infants, α-chymotrypsin plays an essential role in protein digestion. One study reported that α-chymotrypsin cleaves peptides to a similar extent as trypsin, highlighting its importance for breast milk protein digestion (77). Therefore, activity of α-chymotrypsin should be considered in pancreatin (relative to trypsin activity), when designing *in vitro* digestion studies.

Other proteolytic enzymes include elastase and carboxypeptidase, although data on their activity are scarce. Reported values include elastase concentration of 0.6 μg/mL in preterm infants GA 23–30 weeks (78) and carboxypeptidase concentration of 2.9 nmol/min/mL in term infants at one month of age (69). This indicates relatively low activity of these enzymes during early infancy, which was also suggested in a review by Bourlieu et al. (44). Therefore, these enzymes are not usually included in the *in vitro* intestinal digestion studies.

#### 2.3.4 Intestinal glycolytic enzymes

Another enzyme produced by the pancreas is amylase, which is responsible for starch digestion (79). No amylase activity has been detected in the duodenal fluid of term (69) and preterm (72) newborns. Some studies have reported small amounts of amylase in newborn infants, although exact values were not always provided (48) with one study reporting 1.2–5.99 U/mL (47). These findings indicate that amylase activity is negligible in newborn infants, and literature evaluating its activity in older infants remain limited. Moreover, as breast milk contains no starch, amylase is generally considered unimportant in *in vitro* studies evaluating breast milk digestion. Amylase is present in pancreatin, so studies using pancreatin for intestinal digestion inherently include amylase, although its activity is often overlooked in studies evaluating digestion of breast milk. Only a few *in vitro* studies, such as a study evaluating infant digestion of rice-based meals in 6-month-old infants, reported the amylase activity at 75 U/mL (20). When digestion of starchy food is evaluated in weaning infant models, it is recommended to include amylase in the digestion model.

#### 2.3.5 Other intestinal enzymes

Lactase is a brush border enzyme responsible for the hydrolysis of lactose. It is detectable from around eight weeks of GA, reaching approximately 30% of adult activity between 26–34 weeks GA (80) and its activity gradually increases until birth (81). Data on lactase activity in infants is limited. Preterm infants have been reported to exhibit lower lactase activity (82, 83). However, lactase activity was shown to increase in preterm infants 26–29 weeks GA after the initiation of oral feeding, reaching approximately 14.1 U/L within one week (84). This activity is comparable to that observed in children aged 10 months to 14 years (85). Lactase activity has been reported to range from 7.9–19 μmol/g of jejunal tissue in newborns (86) and 25 U/g protein when measured in exfoliated intestinal epithelial cells collected from the lumen in infants aged 2–5 months with lactose intolerance (87). Despite the key role of lactase in the metabolism of breast milk lactose, it is often excluded from *in vitro* digestion models, as these systems typically do not replicate the intestinal brush border. Instead, the models focus on pancreatic enzymes for which activities are better characterised. In studies where breast milk lactose metabolism is of interest, relevant amount (and activity) of lactase enzyme should be incorporated into the *in vitro* model to closely reflect physiology of the infant digestion.

Sphingomyelinase (another brush border enzyme) catalyses the hydrolysis of sphingomyelin, a membrane-associated sphingolipid essential for maintaining lipid bilayer structure and function (88). Its activity is pH dependent, with alkaline sphingomyelinase representing the predominant isoform responsible for sphingomyelin metabolism in the intestinal brush border (89). Sphingomyelinase has been detected in meconium (first stool) of term infants, with higher levels reported in preterm infants (23–36 weeks GA) (90). In newborns, alkaline sphingomyelinase activity has been measured at approximately 13 nmol/h/mg protein in small intestinal biopsy samples (91). Sphingomyelinase is typically not included in *in vitro* digestion protocols due to limited characterisation of its activity. Moreover, as mentioned above for lactase, most *in vitro* intestinal models do not usually replicate the enzymatic environment of the intestinal brush border.

#### 2.3.6 Bile and bile salts

Bile facilitates fat digestion by emulsifying dietary lipids and forming micelles, which enhance the activity of lipases and enable absorption of fatty acids (92). Bile salt concentrations in the fasted state have been reported as 3.8 mmol/L in preterm infants (GA 35 weeks) (93) and 3.3 mmol/L in term infants aged 2–7 days (94). Postprandial concentrations were 2.5 mmol/L in preterm infants (35 weeks GA) (93) and 2.2 mmol/L in infants aged 3–72 months (95). In newborns with GA of 26–41 weeks (both term and preterm), bile salt concentrations after birth were approximately 2 mmol/L (96). Notably, concentrations above 6 mmol/L were found to inhibit lipolysis (even in the presence of colipase) (96), which is an important consideration when designing *in vitro* digestion models. *In vitro* digestion studies have employed either a mixture of two bile salts, sodium taurodeoxycholate and sodium glycodeoxycholate, each at 1 mmol/L (14, 17, 54) or bovine bile extract at 3.1 mmol/L (12, 13, 19, 21). These concentrations correspond to the values reported in infants and are an acceptable approximation for simulating bile-mediated lipid digestion *in vitro*.

### 2.4 Small intestinal acidity

Infants generally exhibit slightly lower intestinal pH values compared to adults (97). In newborns, duodenal pH typically ranges from 6 to 7, gradually increasing to 6.5–7.5 in the ileum (97, 98). Post-pyloric aspirates collected postprandially have reported mean pH values of approximately 6.6 in preterm newborns 34 weeks GA (99), in infants 2.5 years old (100), and in children under four years old (101). In early infancy (1–3 months), enteric fluid samples showed duodenal pH values approximately 5.8 to 6.9 among healthy breast-fed infants (102).

*In vitro* digestion studies have used a range of intestinal pH values, including 6.2 (12, 21), 6.5 (11, 14, 54), 6.6 (13) and in some cases as high as 7.5 (17) or 8 (15). Selection of pH value is critical to reflect the infant duodenal environment and optimise pancreatic enzyme activity. Pancreatic enzymes are known to be most active in neutral to slightly alkaline pH, with pancreatic lipase (in presence of colipase) active between pH 6 and 8 (103, 104) and trypsin active between pH 7 and 8 (105). Although the slightly lower duodenal pH observed in infants may reduce trypsin activity, the enzyme remains functional and its activity increases with higher pH, which is associated with postnatal age (46). Thus, optimal pH setting for *in vitro* digestion studies depends on the food and age of the infant studied. For example, higher values (pH 7–8) have been applied when investigating digestion of protein-rich foods such as beef in 6–12 months old infants (17). A pH of approximately 6.5–6.6 is physiologically relevant for *in vitro* intestinal digestion of breast milk in infants up to six months, reflecting median duodenal pH and remaining within the optimal range for key pancreatic enzyme functions. During *in vitro* intestinal digestion experiments, the intestinal pH may fluctuate due to the buffering capacity and compositional changes in the digesta, thus, continuous adjustment of pH is recommended to maintain the target intestinal pH throughout digestion experiments.

## 3 Small intestinal nutrient absorption

Intestinal nutrient absorption refers to the process by which digested nutrients are transported from the intestinal lumen into the bloodstream (106). There are limited data on absorption of nutrients in infants and mainly limited to animal models. Due to the high nutritional demands in early infancy, nutrient absorption is the greatest immediately after birth (5, 6). Evidence also indicates that macromolecular absorption occurs in infants and is more pronounced in preterm than in term infants (107). Following breast milk feeding, preterm infants (31 weeks GA) exhibit higher circulating levels of *α*-lactalbumin compared with term infants, reflecting an adaptive response to their increased nutritional demands (108). Intestinal villi and crypts play a crucial role in enhancing nutrient uptake by increasing the absorptive surface area (109). Brush border, which contains villi and crypts develops between 9–20 weeks GA (5) and some absorptive cells were reported to be fully developed by 22 weeks GA in the human foetuses (110). These findings further suggest that intestinal absorptive function is not fully mature until term. Following the initiation of feeding, villus size increases within 24 h after birth as reported in piglets (111).

Current infant *in vitro* digestion models typically do not consider nutrient absorption. *In vitro* absorption approaches in general include integrating cell-based models such as Caco-2 intestinal monolayers (112), intestinal organoids (113) or advanced technologies such as Organ-on-chip (OoC) (113). Other options include employing dialysis systems to approximate nutrient absorption (114) or tissue based models such as an Ussing chamber (113). Small intestinal absorption models should also mimic brush border enzymes and functions to better reflect the physiological digestion and absorptive functions. However, due to the rapidly changing physiology of the infant small intestine and its immature absorptive functions, these systems require relevant adaptations to better reflect infant absorption physiology.

### 3.1 Intestinal mucus layer

The intestinal epithelium is covered by a mucus layer secreted by Goblet cells, which protects the epithelial surface from mechanical damage, forms a barrier between the tissue and intestinal contents, and facilitates absorption (115). In the infant intestine, mucus secretion begins around ten weeks of gestation, and mucin-encoding genes (mucins are the main glycoproteins in mucus) have been detected in foetuses between 6.5–27 (116) and at 10 weeks of GA (117). The composition of intestinal mucus changes with postnatal maturation (118), as reported in newborn rats (119) and piglets (120), where the mucus is more heterogeneous and permeable compared with that of adult pigs (120). Incorporating mucins or a mucus layer into infant *in vitro* intestinal models, particularly for the studies focused on structure–function relationship and nutrient absorption, could improve physiological relevance by better replicating the intestinal barrier. Given that infant mucus is thinner and more permeable than that of adults, it may also contribute to faster nutrient absorption. However, the limited understanding of infant mucus composition poses a challenge for accurately reproducing the mucosal layer, and thus, most infant *in vitro* digestion models do not completely replicate this component in their design.

## 4 Differences in static and dynamic *in vitro* digestion models

To date, there is limited direct comparison of static and dynamic infant digestion specifically for human milk. Ménard et al. (11) used a dynamic intestinal model to study the digestion of human milk and observed casein and whey proteins proteolysis patterns comparable to those reported in static intestinal digestion study evaluating digestion of infant formula (74). A study evaluating hydrolysis of major proteins in infant formula using static model found that no intact proteins remained after just five minutes of intestinal digestion (13). In contrast, a study on infant formula using dynamic digestion model showed that proteolysis increased gradually after digesta entered the small intestine, remained relatively constant for most of the intestinal phase, and then increased slightly during the final 60 min of digestion (21). In the static digestion study, free fatty acid (product of lipolysis) concentrations increased rapidly during the first 15 min of the intestinal phase and then remained relatively constant until the 90-min endpoint (18). In contrast dynamic study on human milk reported that free fatty acids increased gradually throughout intestinal digestion, reaching their highest levels at 180 min (12). It is important to note that free fatty acids were measured using different methods in the mentioned studies.

Results from these studies suggest that adding the whole dose of enzymes at the beginning of digestion leads to rapid protein and lipid hydrolysis, whereas in dynamic digestion models the increase occurs more gradually relative to the gradual enzyme addition (and emptying of contents). The impact of the dynamic model, especially those models considering intestinal motility, would also be beneficial in studies that evaluate dynamic structural changes human milk during digestion (and its impact on rates of nutrient absorption). To the best of our knowledge, limited studies have focused on this aspect. In adult *in vitro* digestion models it is known that food structure is altered to a greater extent in dynamic models compared to static models (121), as dynamic models more closely replicate *in vivo* conditions.

Although dynamic infant models are a step closer to mimic physiological conditions of the infant small intestine (compared to static models), most current models do not fully replicate intestinal motility and absorption. In addition, many dynamic models introduce enzymes gradually at a constant flow rate, which provides a practical approximation of secretions but does not fully capture the variability and regulatory patterns of enzyme delivery that occurs *in vivo*. Therefore, harmonised protocols for both static and dynamic models of infant small intestinal digestion need to be developed.

## 5 Conclusion

Simulating intestinal digestion in infants remains a challenge due to the dynamic and developing nature of their gastrointestinal system. *In vivo* data for the infant small intestinal digestion are limited. Available studies indicate considerable variability between preterm and term infants, across different ages, and between individual infants. Enzyme activities, pH, intestinal motility, transit time clearly change with infants age, yet most current *in vitro* intestinal digestion studies do not specify the age group being modelled. This underscores the need for *in vitro* digestion models that reflect the developmental stage of the infant. Additionally, current static *in vitro* digestion models tend to overestimate the speed of nutrient hydrolysis due to immediate enzyme addition, whereas dynamic models allow for more gradual and physiologically relevant nutrient breakdown. However, dynamic models still require significant improvement to better replicate intestinal motility, absorption, and *in vivo* enzyme regulation.

For studies focusing on breast milk digestion, enzymes that aid hydrolysis of lipids and proteins are critical. Thus, enzyme selection should take into consideration nutrient composition of the milk, infant age of interest, and purpose of the digestion study. Infants also possess distinct lipolytic enzymes, such as BSSL, PLRP1 and PLRP2. However, physiological data on their activity and developmental regulation remain limited. Similarly, evidence regarding some proteolytic enzymes, including *α*-chymotrypsin, elastase, and carboxypeptidase is limited. The lack of physiological data, together with the restricted commercial availability of some intestinal enzymes, such as, e.g., BSSL, PLRP 1,2 frequently leads to their exclusion from infant *in vitro* digestion models. This can reduce physiological relevance of *in vitro* intestinal digestion studies.

Altogether, this mini-review highlights challenges, provides recommendations, and emphasises the need for the development of standardised, age-specific, and physiologically relevant infant *in vitro* intestinal digestion protocols for studying breast milk digestion. Such developments are essential to improve our understanding of nutrient digestion and availability during early infancy.
